# DPP-IV Inhibitory Peptide against In Vitro Gastrointestinal Digestion Derived from Goat’s Milk Protein and Its Activity Enhancement via Amino Acid Substitution

**DOI:** 10.3390/foods13172721

**Published:** 2024-08-27

**Authors:** Baoyuan He, Yanhui Lian, Haiyan Xue, Yan Zhou, Yi Wei, Jun Ma, Yalin Tan, Yawen Wu

**Affiliations:** 1 College of Bioresources Chemical & Materials Engineering, Shaanxi University of Science and Technology, Xi’an 710021, China; hebaoyuan@sust.edu.cn; 2 School of Food Science and Engineering, Shaanxi University of Science and Technology, Xi’an 710021, China; lianyanhui1999@163.com (Y.L.); zy18792317301@163.com (Y.Z.); 17393808978@163.com (Y.W.); majun@sust.edu.cn (J.M.); 13893314503@163.com (Y.T.); 15393301916@163.com (Y.W.)

**Keywords:** digestion resistance bioactive peptide, DPP-IV inhibitory peptides, gastrointestinal digests, goat milk protein, structure–activity relationship, hydrophobic force

## Abstract

Goat milk protein can release a variety of bioactive peptides after digestion, while most of them are digested into free amino acids or dipeptides via the GI tract. We investigated the peptide profiles of goat milk protein following in vitro gastrointestinal digestion using LC-MS/MS and identified 683 bioactive peptides, including 105 DPP-IV inhibitory peptides. Among these peptides, ILDKVGINY (IL), derived from β-lactoglobulin, was found to be high in content and resistance to digestion. Herein, we explore the effect of amino acid residue substitution at the second N-terminus on its DPP-IV inhibitory activity. Three 9 polypeptide fragments (peptide IL, IP, and II) were synthesized and subjected to molecular docking and activity analysis. The peptide IL demonstrated the highest affinity for DPP-IV with a binding energy of −8.4 kcal/mol and a moderate IC50 value of 1.431 mg/mL determined based on the Caco-2 cell model. The replacement of specific amino acid residues by Pro and Leu led to an increase in the hydrophobic force interaction between the inhibitor peptide and DPP-IV. The inhibition rates of the three peptides were significantly different (*p* < 0.05). Peptide II containing an Ile residue instead of Leu resulted in a significant enhancement of DPP-IV inhibitory activity, with an IC50 value of 0.577 mg/mL. The GRAVY changes in the three peptides were consistent with the trend of the inhibitory rates. Therefore, the GRAVY of peptides and branch-chain amino acids should be considered in its activity improvement. The present study revealed the presence and activity of DPP-IV inhibitory peptides in goat milk, providing important insights for further investigation of their potential food functionality and health benefits.

## 1. Introduction

The nutritional composition of goat’s milk closely resembles that of breast milk, received much attention, and is often referred to as the “King of milk” [1]. Goat’s milk is rich in protein, and the enzymatic hydrolysis of these proteins can generate different biologically active peptides. Research into these peptides gained popularity in recent years. The hydrolysis of goat milk proteins produces various physiologically active peptides, including antioxidant, immunologically active, and ACE inhibitory peptides, and more [2]. The structure of peptides is closely related to their physiological activities.

Dipeptidyl peptidase-IV inhibitors are a new type of antidiabetic drug. DPP-IV inhibitors received increasing attention in recent years. Food-borne DPP-IV inhibitory peptides derived from natural proteins offer several advantages, including high safety, enhanced human absorption, and selective blood sugar reduction. These peptides emerged as promising functional food ingredients with significant potential applications. DPP-IV contains two structurally symmetrical chains. On the outside of the enzyme are structural domains containing α/β catalysis, structural domains containing β-helices, and a boundary layer region. S1, S2, and S3 are three binding pockets, and the inhibitor bound at the binding pocket is a competitive inhibitor [3].

The DPP-IV plays a crucial role in glucose metabolism. After food intake, blood sugar levels will increase, and intestinal L-cells in the digestive tract secrete two types of incretins: GLP-1 and GIP. There are two possible effects of an increase in blood sugar levels. First, it inhibits glucagon production. Secondly, it stimulates the islet β cells to secrete more insulin, which helps to stabilize blood sugar balance [4]. DPP-IV, a serine protease, is expressed in multiple tissues and can hydrolyze alanine (Ala) in GLP-1, resulting in its deactivation. Additionally, it can degrade two amino acids located at the end of the GIP. Therefore, DPP-IV inhibitors primarily function by inhibiting the enzymatic activity of DPP-IV, thereby regulating GLP-1 and GIP levels in patients. This leads to an increase in insulin secretion and ultimately facilitates the reduction in blood sugar levels through the action of peptides with DPP-IV inhibitory properties [5].

Whey protein has a hypoglycemic effect, which may be due to the DPP-IV inhibitory peptide contained in it [6]. Researchers injected diabetic mice with different doses of donkey blood-derived DPP-IV active peptide inhibitor and found that the active peptide exhibited a notable reduction in postprandial blood glucose levels in diabetic mice. Furthermore, it demonstrated a significant improvement in glucose tolerance in mice [7]. Food-borne peptides that inhibit DPP-IV emerged as a significant approach for adjunctive therapy in the future; therefore, the application of peptides in drugs is a novel avenue for drug research and development. The Caco-2 cell monolayer model is a widely used model for investigating the absorption of drugs and other substances in the small intestine [8]. Nowadays, it is also extensively employed for the assessment of intestinal absorption of various active peptides, including those associated with blood pressure. The higher quantitative and qualitative uptake of the nanoemulsion by Caco-2 cells indicated good intestinal absorption and enhanced therapeutic activity of nisoldipine. This ultimately leads to significantly increased bioavailability and antihypertensive effects of the nisoldipine nanoemulsion in comparison to its dispersion [9]. Currently, there is a limited body of research on bioactive peptides associated with goat milk and goat whey, indicating the need for further development and investigation in this area. The identification of potential active peptides through structure–activity relationship analysis is important for facilitating the development of new DPP-IV inhibitory peptides with enhanced activity.

In this study, the digestive juice was analyzed after simulated gastrointestinal digestion for 30 min in vitro using LC-MS/MS. The obtained mass spectrum data and a map of the digestive juice were used to analyze and identify the polypeptide fragments generated in the hydrolysate. The identification process involved referencing the polypeptide database available at http://mbpdb.nws.oregonstate.edu/, accessed on 21 July 2023. Two additional peptides with DPP-IV inhibitory activity were synthesized using ILDKVGINY as a reference. Subsequently, computer simulation was conducted using molecular docking. Finally, the DPP-IV inhibitory activities of the three peptides were tested using Caco-2 cells to verify the structure–activity relationship of these peptides.

## 2. Materials and Methods

### 2.1. Chemicals

ILDKVGINY, IIDKVGINY, and IPDKVGINY were provided by Nanjing Peptide Valley Biotechnology Co., Ltd. (Nanjing, China). The human colorectal adenocarcinoma Caco-2 cell line was purchased from Wuhan Pricella Biotechnology Co., Ltd. (Wuhan, China). Trypsin-EDTA digest, DMEM high glucose medium, fetal bovine serum, and dimethyl sulfoxide (DMSO) were purchased from Beijing Solarbio Science & Technology Co., Ltd. (Beijing, China). DMEM high glucose medium, non-essential amino acids were provided by Beijing Zhongke Maichen Technology Co., Ltd. (Beijing, China). 3,4,5-dimethylthiazole-2,5-diphenyl-tetrazolium bromide (MTT) was purchased from, Beijing Boao Toda Technology Co. (Beijing, China). All other high-purity reagents were purchased from Sinopharm Chemical Reagent Co. (Beijing, China).

### 2.2. Simulated Gastrointestinal Digestion

Goat milk was subjected to simulated gastrointestinal digestion according to the in vitro digestion protocol described by Xue et al. [10]. Artificial gastric fluid from goat milk was mixed in a 1:1 ratio, followed by the addition of 120 μL CaCl_2_(H_2_O)_2_. The pH of the resulting solution was adjusted to 2.0 using HCl. The solution was then divided into centrifuge tubes, with each tube containing 20 mL of the solution. The tubes were placed in a water bath (FYDKH3, Shanghai Hengyi Scientific Instrument Co., Shanghai, China) and subjected to shock for five min for pre-heating. Subsequently, pepsin was added and dissolved in simulated gastric fluid (SGF). After preheating, 1 mL enzyme solution was added to each tube to initiate the in vitro digestion process. Digestion time intervals were established at 0, 10, 30, 60, 90, and 120 min. Additionally, the pH of the digestion solution was adjusted to 8.0, using NaOH solution to deactivate the enzyme following digestion. After gastric digestion for 60 min followed by enzyme inactivation, an artificial intestinal solution was added at a ratio of 1:1. The subsequent experimental procedures were identical to those previously described. The pH of the solution was adjusted to 7.0. Digestion time intervals were set at 0, 0.5, 5, 10, 30, and 60 min. After digestion, the resulting solution was subjected to heat treatment in a water bath set at 100 °C for 5 min. This step is performed to deactivate the enzyme. Subsequently, each tube containing the sample was stored in a refrigerator at −20 °C for freezing.

### 2.3. Detection of Milk Protein Hydrolysate by LC-MS/MS

LC-MS/MS was determined as described by Sun et al. [11]. Liquid chromatography-tandem mass spectrometry (LC-MS/MS) was used to analyze the hydrolysate of gastrointestinal co-digestion for 30 min and obtain the digested peptidomics of the proteins. The hydrolysate was firstly dissolved with the sample dissolution solution (0.1% formic acid, 2% acetonitrile) and then centrifuged (HC-3018R, Anhui Zhongke Zhongjia Scientific Instrument Co., Hefei, China) at 13,200 rpm for 20 min at 4 °C, and the supernatant was extracted for mass spectrometry identification. The 6-step linear gradient elution (A: 0.1% formic acid, B: 0.1% formic acid, 80% CAN) was performed: 0–5 min, 5% B; 5–45 min, 45% B; 45–50 min, 90% B; 50–55 min, 90% B; and 55–65 min, 5% B. The chromatographic system consisted of a capture column (300 μm idx 5 mm, Acclaim PepMap RSLC C18, 5 μm, 100 Å, (160454, Thermo, Waltham, MA, USA)) and an analytical column (Acclaim PepMap 75 μm × 150 mm, C18, 3 μm, 100 Å (160321, Thermo, Waltham, MA, USA).

The separated peptides were directly introduced into a Thermo Scientific Q Exactive mass spectrometer (Thermo, Waltham, MA, USA) for real-time detection. Primary mass spectrometry was performed with the following specific parameters: a resolution of 70,000, an AGC target of 3e6, a maximum IT of 100 ms, and a scan range of 350 to 1800 m/z. Secondary mass spectrometry was conducted with a resolution of 17,500, AGC target of 5e4, maximum IT of 120 ms, and TopN value of 20. In addition, the NCE/stepped NCE was set to 27. The parameters were resolution: 17,500; AGC target: 5e4; maximum IT 120 ms; TopN: 20; and NCE/stepped NCE: 27. The initial mass spectra were processed using MM file conversion software (Msconvert version 2.0), resulting in their conversion into the MGF format. Subsequently, the spectra were extracted from the MGF file using MASCOT (http://www.matrixscience.com/, accessed on 20 March 2023) to search the UniProt database and obtain results. Active peptides identified by LC-MS/MS were analyzed for peptide activity using the Milk-Based Peptide Activity Database (MBPDB, http://mbpdb.nws.oregonstate.edu/, accessed on 21 July 2023). Solid phase synthesis was taken to synthesize the desired DPP-IV inhibitory peptide (Nanjing Peptide Valley Biotechnology Co., Ltd., Nanjing, China).

### 2.4. Molecular Docking

AutoDock Vina (version 1.1.2) was used as the molecular docking software for peptide fragment in the present investigation. Please retrieve the three-dimensional structure file of DPP-IV from the RCSB database. DPP-IV was associated with the peptide fragments ILDKVGINY(IL), IPDKVGINY(IP), and IIDKVGINY(II). PYMOL software (version 4.3.0) (https://pymol.org/, accessed on 10 September 2023) was used to extract the native ligand from the protein structure, eliminate hydration, and eliminate organic components. AutoDock (version 1.1.2) is a software tool commonly employed in the determination of twist bonds in small peptide molecules, hydrogenation of proteins, charge calculation, and atomic-type assignment. After establishing a connection with Vina, force analysis and visualization of three-dimensional and two-dimensional angles were conducted using Discovery Studio 2019 software. Owing to its symmetrical structure, DPP-IV only requires the utilization of one of its chains for docking, thereby reducing the computational workload involved in the docking process.

### 2.5. Determination of the Optimal Concentration of DPP-IV Inhibitory Peptide Using Caco-2 Cells

Cells (Wuhan Pricella Biotechnology Co., Ltd., Wuhan, China) were cultured under standard conditions at 37 °C and a CO_2_ concentration of 5%. Caco-2 cells cultured for a period of 2–3 days were subjected to trypsin (Beijing Solarbio Science & Technology Co., Ltd., Beijing, China) treatment. The cells were seeded into a 96-well plate at a density of 5 × 10^4^ cells/well. Subsequently, 100 μL of polypeptide solution was added at concentrations of 0.01 mg/mL, 0.1 mg/mL, 0.2 mg/mL, and 0.5 mg/mL. After aspirating the polypeptide solution, PBS solution was added for cleaning. To initiate the experiment, 20 μL MTT (Beijing Boao Tuoda Technology Co., Ltd., Beijing, China) reagent was added to each well. The well plate was then incubated at 37 °C for 4 h to ensure that the incubation was carried out in the dark. Following this, the liquid in the well plate was carefully removed, and 150 μL of DMSO reagent was added. The plate was again incubated at 37 °C for 30 min. To ensure complete dissolution of the crystals, the plate was shook for 10 min. Finally, the absorbance of the substance was measured at a wavelength of 490 nm (EV-2200, Shanghai Mepda Instrument Co., Shanghai, China) [12]. Calculate and analyze using the provided formula.

(1) $$cytoactive\left(\%\right)=\frac{{OD}_{d490}-{OD}_{s490}}{{OD}_{k490}}\times 100\%$$

where d stands for control group; S stands for experimental group; and K stands for blank group.

### 2.6. Verification of DPP-IV Inhibitory Activity

Validation of the inhibitory activity of DPP-IV was carried out with slight modifications in reference to the method of Wu et al. [13]. The cells were inoculated into a black 96-well plate at a density of 5 × 10^4^ cells per well. After three days of culture, the culture medium was aspirated and the plate was washed with phosphate-buffered saline (PBS). Adding 100 μL of sitagliptin at various concentrations (10^−5^, 10^−4^, 10^−3^, 10^−2^, and 10^−1^ mg/mL), three polypeptide solutions were synthesized: ILDKVGINY, IPDKVGINY, and IIDKVGINY (at concentrations of 10^−5^, 10^−4^, 10^−3^, and 10^−4^). The excitation wavelength was set to 350 nm, the generation wavelength was set to 480 nm in the microplate analyzer, and the fluorescence intensity was measured.

### 2.7. Statistical Analysis

The amino acid sequence was analyzed using liquid chromatography mass spectrometry/mass spectrometry (LC-MS/MS) sequential software and PEAKS 7.0. The data obtained were visualized using GraphPad Prism 6.01 software and analyzed using SPSS 22.0 software for significance analysis (*p* < 0.05) via one-way analysis of variance (ANOVA).

## 3. Results

### 3.1. Distribution of Peptides after Gastrointestinal Digestion

**Figure 1:**
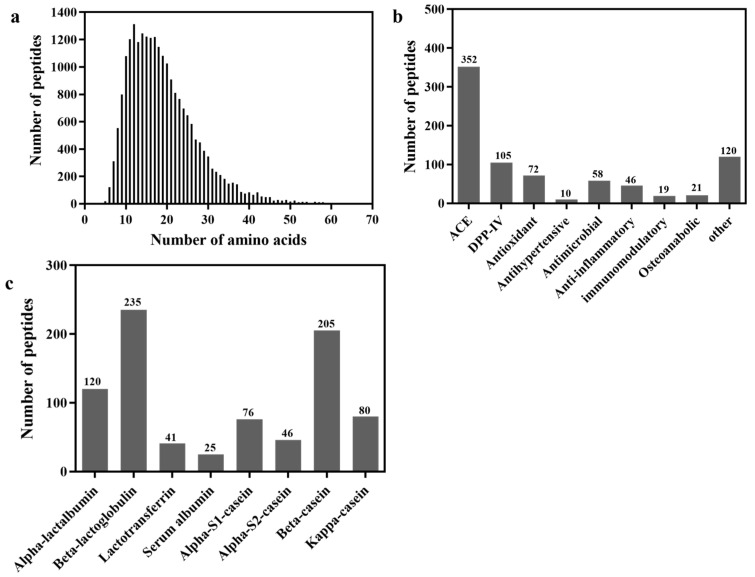
Hydrolyzed peptide segment distribution diagram. (**a**) Distribution of peptide lengths; (**b**) distribution of peptide activities; and (**c**) distribution of peptide source proteins.

Goat milk was enzymatically digested using in vitro gastrointestinal digestion to generate biologically active peptides, and the extent of peptide degradation was assessed. Figure 1a illustrates the length distribution of hydrolyzed peptides, which exhibited a normal distribution pattern. The majority of the peptides were concentrated within the range of 10–20 lengths. Figure 1b illustrates a variety of active peptides that are frequently encountered. For instance, various types of bioactive peptides were identified, including ACE inhibitory peptides, DPP-IV inhibitory peptides, antioxidant peptides, and immunoactive peptides. As depicted in Figure 1b, the hydrolysate obtained after a 30 min period of combined gastrointestinal digestion exhibited the highest concentration of ACE-inhibitory peptides, reaching a remarkable count of 352. Additionally, a substantial number of DPP-IV inhibitory peptides were identified, totaling 105 peptides. As depicted in Figure 1c, the peptides evaluated for their activity consisted of 235 peptides derived from β-lg and 205 peptides derived from α-CN.

### 3.2. Screening of DPP-IV Inhibitory Peptides

As indicated in Table 1, a total of eight peptides exhibiting DPP-IV inhibitory activity were obtained from β-CN, four from κ-CN, five from α-La, eight from β-Lg, three from milk serum albumin, and two from lactoferrin. Among them, the DPP-IV inhibitory peptide fragment derived from milk serum albumin may be rapidly degraded in vivo due to low structural stability, leading to loss of activity. Analysis of the peptides with DPP-IV inhibitory activity revealed that ILDKVGINY, VGINYWLAHK, and ILDKVGINYWLAHK shared the same fragment structure, and all three physiologically active peptides displayed DPP-IV inhibitory activity, including the peptide ILDKVGINY, which contains lysine (K) and aspartic acid (D); and it was shown that aspartic acid decreases the cleavage efficiency of trypsin. Certain amino acids within peptide sequences may change the DPP-IV inhibitory activity of peptide fragment.

### 3.3. Analysis of the Nature and Structure of IL, IP, II

Using the ILDKVGINY peptide, which possess DPP-IV inhibitory activity, as a synthetic reference, we considered the substitution of amino acids at the second position of the N-terminus because the N-terminus is already the most hydrophobic amino acid, isoleucine (Ile). The isoelectric point and molecular weight were evaluated using EXPASY-Compute PI/MW (http://web.expasy.org/compute_pi/, accessed on 20 september 2023). The average hydrophilicity of peptides (GRAVY) can be predicted by http://web.expasy.org/protparam/, accessed on 22 september 2023. The net charge and hydrophobicity of the peptide were analyzed by http://www.tulane.edu/~biochem/WW/PepDraw/index.html, accessed on 22 september 2023.

In conjunction with Supplementary Materials, analysis of the nature of the peptide fragments showed that the number of amino acids in IL, IP, and II was nine. The polyalphabetic representation of IL was H_2_N-Ile-Leu-Asp-Lys-Val-Gly-Ile-Asn-Tyr-COOH, the polyalphabetic representation of IP was H_2_N-Ile-Pro-Asp-Lys-Val-Gly-Ile-Asn-Tyr-COOH; and that of II was H_2_N-Ile-Ile-Asp-Lys-Val-Gly-Ile-Asn-Tyr-COOH. The isoelectric points, hydrophilic residue ratios, extinction coefficients, and average hydrophilicities of the three synthesized polypeptides were the same. The three synthetic peptides differed in molecular weight and GRAVY; the molecular weights were not very different, and the molecular weights of IL and II were the same, whereas the GRAVY values of the peptides differed more, and the GRAVY values of the peptides were calculated as the sum of the hydrophilic values of all amino acids divided by the number of amino acid residues in the sequence. The three synthesized peptides exhibited an equal distribution of hydrophilic residues, but displayed a significant disparity in GRAVY values. This discrepancy suggests that there is variation in hydrophilicity among the three synthesized peptides. This difference in hydrophilicity may be attributed to the structural characteristics of peptide segments.

**Figure 2:**
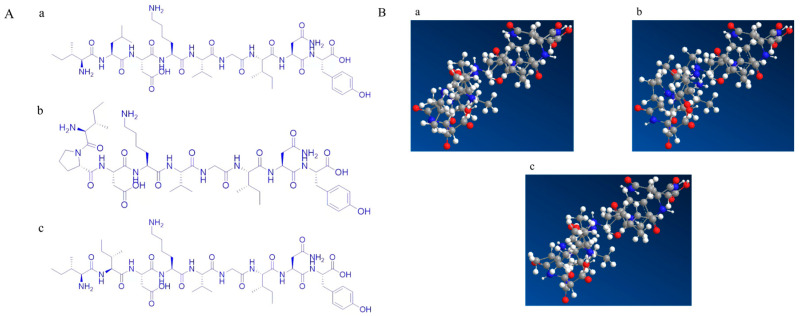
Structure diagram of synthetic peptide segment. (**A**) Plane structure diagram of the synthetic peptide segment. (**a**) ILDKVGINY; (**b**) IPDKVGINY; and (**c**) IIDKVGINY. (**B**) Stereo structure diagram of the synthetic peptide segment. (**a**) ILDKVGINY; (**b**) IPDKVGINY; and (**c**) IIDKVGINY.

The stereo structures of the peptides were dissected, and it can be seen from Figure 2 that the three peptides differed greatly in the second amino acid. Neither IL nor II have a cyclic structure, but the planar structure at the second amino acid of IP has a cyclic structure because proline has a special cyclic structure. Four amino acids possess a cyclic structure in their R group, namely phenylalanine, tyrosine, tryptophan, and histidine. Although proline also has a cyclic structure, its amino group is located in the cyclic structure, not the R group, so it is not an “amino acid with a cyclic structure in the R group”. In addition, the side chains of proline cannot form hydrogen bonds; ILs and II do not have a ring structure, but isoleucine and leucine on the second amino acid from the N-terminus of both are isomers. Leucine has one chiral carbon atom, isoleucine has two chiral carbon atoms, and the rotation of the chiral carbon atoms results in different conformations.

### 3.4. Results of Molecular Docking of Peptides with DPP-IV Enzymes

**Figure 3:**
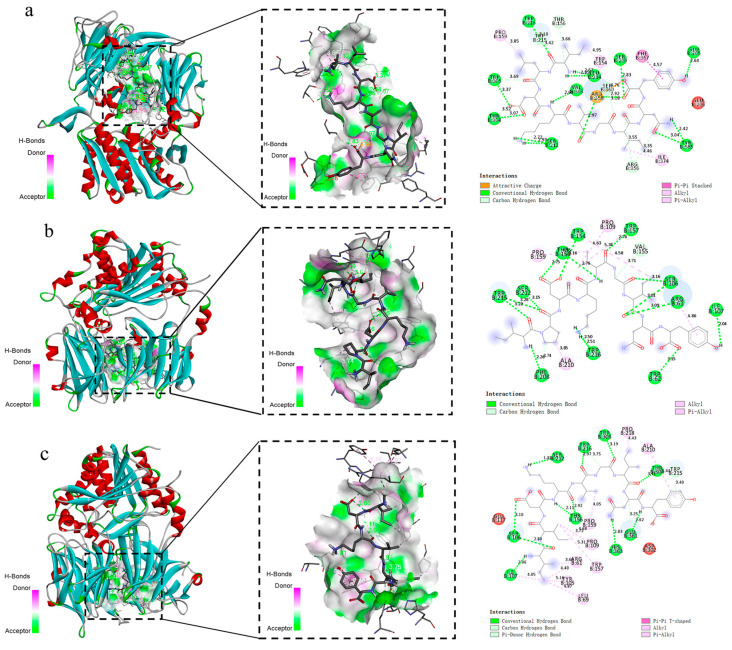
Molecular docking diagrams of peptide segments with the DPP-IV enzyme. (**a**) Three-dimensional plan view and two-dimensional plan view of ILDKVGINY interaction with the DPP-IV enzyme. (**b**) The 3D plan view and 2D plan view of IPDKVGINY interaction with the DPP-IV enzyme. (**c**) Three-dimensional plan view of IIDKVGINY interaction with the DPP-IV enzyme. In the two-dimensional plan, the green dashed lines indicate hydrogen bonds, light green dashed lines indicate carbon–hydrogen bonds, and pink dashed lines indicate hydrophobic forces.

Analysis of Figure 3 and Table S1 reveal 17 binding sites of the IL to the DPP-IV enzyme. Except for one electrostatic force of 4.76 Å that binds to Arg residue 358 of the DPP-IV enzyme, the remaining 24 linkages are hydrogen bonds (Figure 3a). There are 13 binding sites of IP to DPP-IV, which is less than that of IL to DPP-IV. There were six hydrophobic interactions with the DPP-IV enzyme, and the remaining fourteen were hydrogen bonds. Pro is unique in that its side chain cannot form hydrogen bonds (Figure 3b). II has 18 binding sites with the DPP-IV enzyme, which is one more than that of the IL with the DPP-IV enzyme. Ten hydrophobic interactions occur with the DPP-IV enzyme, and the remaining 12 bonds are hydrogen bonds. The results show that DPP-IV can form hydrogen bonds with the IL, and the remaining 24 bonds are hydrogen bonds (Figure 3c). The results show that the binding energies are between the DPP-IV enzyme and the synthesized peptides, IL, IP, and II, −8.4 kcal mol^−1^, −8.3 kcal mol^−1^, and −7.7 kcal mol^−1^, respectively. Generally, if the binding energy of a ligand to a target protein is less than −5, it indicates that the binding between the protein and the small molecule is stable. Therefore, the binding energies of the ligands IL, IP, and II to the DPP-IV enzyme are strong. The enzyme had a strong affinity between the larger absolute value of binding energy, indicating a stronger affinity between the synthesized peptide and the DPP-IV enzyme. As shown by the results of molecular docking, IL had the strongest inhibitory activity against DPP-IV, IP had the second strongest inhibitory activity, and II had the weakest inhibitory activity.

### 3.5. Effect of DPP-IV Inhibitory Peptides on Cell Viability of Caco-2 Cell

**Figure 4:**
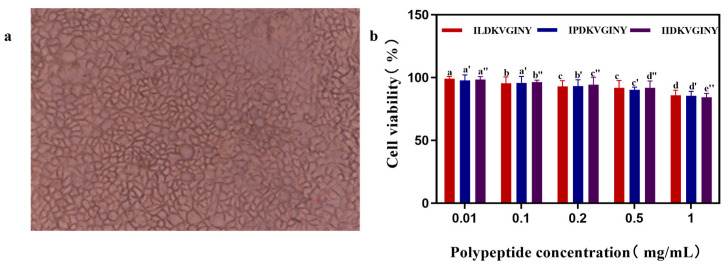
Effect of different peptide concentrations on Caco-2 cell activity. (**a**) Pictures of Caco-2 cell growth. (**b**) Effect of synthetic inhibitory peptide concentration on cell activity. Note: different letters for the same sample indicate highly significant differences in the data (*p* < 0.05).

As shown in Figure 4a, the growth of Caco-2 cells was uniform, the cell morphology was completely and tightly connected, and the cell density was above 90%. As shown in Figure 4b, cell activity decreased gradually with an increase in peptide concentration. There was no significant difference (*p* > 0.05) in cell activity when the peptide concentration was increased from 0.01 mg/mL to 0.1 mg/mL, despite the decrease in cell activity. Under the culture conditions of a peptide concentration of 0.2 mg/mL as well as 0.5 mg/mL, there was no significant difference in cellular activity, but the cellular activity was significantly reduced compared to that at 0.1 mg/mL (*p* < 0.05), and when the peptide concentration was 0.5 mg/mL, the cellular activities of IL, IP, and II cells were 91.91 ± 5.763%, 91.88 ± 5.385%, 91.88 ± 5.385%, 91.88 ± 5.385%, and 90.34 ± 2.08%. The reduction in cellular activity was more pronounced when the peptide concentration was 1 mg/mL. The IL, IP, and II cellular activities were 86 ± 3.911%, 84.46 ± 2.928%, and 85.51 ± 3.52%, respectively. Therefore, when determining the concentration of the synthetic peptide, it was necessary to control the peptide concentration to less than 1 mg/mL.

### 3.6. Results of In Vitro DPP-IV Inhibitory Activity Assay in Caco-2 Cells

**Figure 5:**
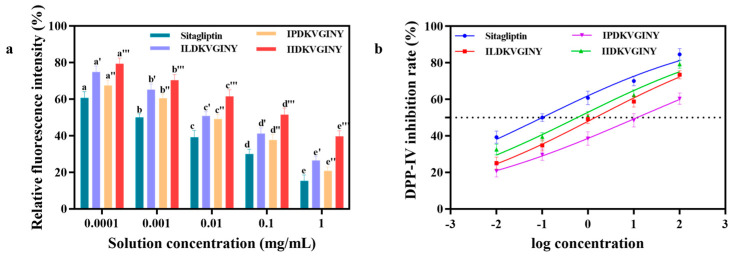
Determination of DPP-IV inhibitory activity of synthetic peptide. (**a**) Comparison of fluorescence intensity of ILDKVGINY, IPDKVGINY, and IIDKVGINY. Note: different letters for the same sample indicate highly significant differences in the data (*p* < 0.05). (**b**) IC50 values of sitagliptin, ILDKVGINY, IPDKVGINY, and IIDKVGINY.

As shown in Figure 5a, with an increase in polypeptide concentration, the relative fluorescence intensity decreased significantly (*p* < 0.05), indicating that the synthesized polypeptide had an obvious inhibitory effect on DPP-IV. When the polypeptide concentration was 1 mg/mL, the DPP-IV inhibition rates of IL, IP, and II were 73.44 ± 2.23%, 60.37 ± 3.04%, and 79.20 ± 2.20%, respectively, and the IC_50_ values of sitagliptin, polypeptide IL, IP, and II were 0.099 mg/mL, 1.431 mg/mL, 11.73 mg/mL, and 0.577 mg/mL, respectively. As can be seen from Figure 5b, the synthetic peptide has an obvious DPP-IV inhibitory effect, and the graph of the effect is positively correlated. A comprehensive analysis showed that the synthetic peptide had an obvious DPP-IV inhibitory effect. The DPP-IV inhibitory effect of II was the strongest, followed by that of IL, and the DPP-IV inhibitory effect of IP was the weakest.

## 4. Discussion

Food proteins contain potential functional active peptide fragments that can be released via enzymatic or gastrointestinal hydrolysis. They produce a diversity of active peptide hydrolysates under different hydrolysis conditions. Herein, LC-MS/MS were used to identify peptide profiles in the simulated gastrointestinal hydrolysis of skim goat milk. The possible active peptides were further screened by matching them with the milk peptide database. It was found that a high amount of ACE inhibitory peptides were produced under in vitro digestion, probably because there are many potential ACE inhibitory peptide sequences in goat milk protein as well, as typsin is more likely to be hydrolyzed in the corresponding peptide by coincidence. Meanwhile, a large amount of DPP-IV inhibitory peptides were also found in in vitro digests (Figure 1b). Trypsin, a serine protease, specifically cleaves C-terminal peptide bonds with lysine and arginine residues. However, trypsin cleaves lysine relatively inefficiently, resulting in the production of incompletely cleaved peptides [26]. This may explain why lysine is not completely cleaved by trypsin in Table 1.

DPP-IV cleaves enteroglucagon-like peptide 1 (GLP-1) and glucose-dependent insulinotropic polypeptide (GIP) along with other enteroglucagon-like peptides, resulting in a reduction in their insulinotropic activity. DPP-IV inhibition preserves the proinsulin activity of GLP-1 and GIP, thus enhancing glucose homeostasis in diabetes. Synthetic pharmacological inhibitors of DPP-IV are used to prolong the half-life of active GLP-1 and GIP [27]. Sitagliptin, rigliptin, vigliptin, saxagliptin, and alogliptin are synthetic DPP-IV inhibitors currently in use [28]. These inhibitors can enhance the function of GLP-1 and GIP, but they may also cause undesirable symptoms, such as headache and fatigue. Therefore, there is a growing desire to discover novel DPP-IV inhibitors as alternatives. Some studies found that whey proteins increase the GLP-1 receptor [29], while other researchers discovered that the rapid increase in branched-chain amino acids in plasma after consuming whey stimulates GLP-1 secretion [30]. The peptide used in this experiment, ILDKVGINY, is derived from β-lactalbumin and is expected to enhance GLP-1 receptor activation. It is reported that the second position of the N-terminus of DPP-IV inhibitory peptides is typically characterized by the presence of either Pro or Ala, because DPP-IV can recognize and cleave Pro or Ala at this position [23]. Considering that Pro has a special cyclic structure, LPDKVGINY was selected as the second peptide segment to study the structure–activity relationship. This activity can be further enhanced by the inclusion of hydrophobic amino acids at either the N- or C-terminal position [31]. Iso leucine (Ile) and leucine (leu) are isomers, so IIDKVGINY was synthesized as the third peptide to determine the effect of amino acid choice on the peptide inhibitory activity.

DPP-IV has two main active pockets: hydrophobic pocket and charged pocket [32]. The hydrophobic pocket S1 consists of several residues, such as Tyr547, Ser630, Tyr631, Val656, Trp659, Tyr662, Tyr666, Val711, and His740 [33,34], Ser630, and His740 participate in the formation of the catalytic triad [35], while charged pocket S2 consists of Arg125, Glu205, Glu206, Ser209, Phe357, Arg358, Tyr662, and Asn710 residues [33,34]. This region is the active site of DPP-IV and binding to this region decreases its activity. In addition, the S1 bag has a narrow gap, which preferentially binds to small hydrophobic inhibitors, while the S2 bag is wider than the S1 bag by binding compounds through salt bridges [3]. Therefore, S2 can hold a larger inhibitor than S1. The DPP-IV inhibitory peptides synthesized in this article are all nine peptides that are large inhibitors in size. Molecular docking revealed that the main peptides were located in the binding site region of S2. After conducting molecular docking, the binding energy between the DPP-IV enzyme and the synthesized peptide IL was −8.4 kcal mol^−1^, indicating a stronger interaction with DPP-IV, while, the affinity of peptides IP and II are less than peptide IL. These could be reflected by a decrease in hydrogen bond in the molecular docking results.

In the process of in vitro digestion, potential active peptide fragments harboring in food proteins may be degraded by digestive enzymes, but the peptide fragment selected in this study still has DPP-IV inhibitory activity after digestion, which is a digestion-resistant peptide fragment with a molecular weight of about 1kDa. Some reported that DPP-IV inhibitory peptides tend to be active peptides with 2–8 amino acid residues and a molecular weight of less than 1 kDa [36]. The DPP-IV inhibition and metabolic stability of casein-derived peptide Val-Pro-Tyr-Pro-Gln (VPYPQ) and its fragments, and their release from casein after hydrolysis were studied in reference [32]. The results show that VPYPQ was the strongest DPP-IV inhibitory peptide, with an IC50 value of 41.45 μM, which may be due to its two internal Pro residues at positions 2 and 4. The peptides with hydrophobic amino acids at the N-terminal position were discovered with high DPP-IV inhibitory activity [23]. It is also reported that the increase in DPP-IV inhibitory activity may be due to hydrophobic amino acids such as leucine (Leu), isoleucine (Ile), and phenylalanine (Phe), which are located at the N-terminal position of the peptide and can interact with the hydrophobic pocket of the DPP-IV active site [37]. Three 9 peptides synthesized here have the same hydrophobic amino acids (Leu) residue at the N-terminal and differed from each other only by one amino acid at position 2. We investigated the substituting of Pro by Leu reside at the second position of the N-terminus; there was no significant difference in the results of molecular docking. Both of them have a good affinity for DPP-IV. However, the inhibitory activity verification based on the Caco-2 cell showed that the inhibition rate of the peptide after replacement did not increase, but significantly decreased. Therefore, we should not only consider the influence of amino acid substitution, but also pay more attention to the influence of secondary structural changes. After replacing Leu at the second N-terminal with Ile, the molecular docking results show that its affinity activity decreased. The characters of three synthesized peptides mainly differ at GRAVY, which indicated the average hydrophilic. The GRAVY of peptide IL, IP, and II were 0.53, −0.07, and 0.61, respectively. These are consistent with the trend of the DPP-IV inhibitory rate. By analyzing the connection bonds, the II fragment had 10 more hydrophobic forces than the IL fragment, and the number of hydrogen bonds was 12 fewer. Therefore, the enhancement of the II fragment’s inhibitory rate might be related to the increase in hydrophobic forces, but more experiments are still needed. In addition, Ile is a branch chain amino acid, which may play a role in the improvement of the inhibitory rate. However, these conclusions need to be precisely verified by a large number of experiments.

## 5. Conclusions

Following an in vitro simulation of goat milk protein digestion, a total of 828 peptides were identified through LC-MS/MS analysis, comprising 683 bioactive peptides and 105 DPP-IV inhibitory peptides. Among these, fragment ILDKVGINY was discovered to exhibit DPP-IV inhibitory activity, originating from β-lactoglobulin and retaining its activity following digestion. It was found that replacing Leu at the second N-terminal site (peptide IP) with Pro did not enhance the affinity of the peptide for DPP-IV; instead, it resulted in a lower inhibition rate. However, replacing Leu at the second N-terminal site (peptide II) with Ile slightly decreased the peptide’s affinity for DPP-IV but increased the hydrophobic force and inhibitory activity. As a result, the role of amino acids with branched chains should be considered in the design of DPP-IV peptide inhibitors, in addition to the hydrophilicity and hydrophobicity of peptides, when enhancing the activity of DPP-IV inhibitory peptides. This study provides recommendations for the screening of digestion-resistant bioactive peptides and the activity improving of DPP-IV inhibitory peptides derived from food proteins.

## Figures and Tables

**Table 1:** DPP-IV Inhibitory active peptide.

Source Peptide Fragment	Active Peptide Fragment	Source Protein	Serial Number
**YPVEPF**TESQS	YPVEPF [14,15]	Beta-casein	129–134
EP**VLGP**VR	VLGP [16]	Beta-casein	212–215
V**LPVPQ**KVVP	LPVPQ [14]	Beta-casein	186–190
NS**LPQ**NILPLT	LPQ [17]	Beta-casein	85–87
LH**LPLPL**V	LPLPL [14,18]	Beta-casein	150–154
LH**LPL**PLV	LPL [18]	Beta-casein	150–152
**FLQP**EIMGVP	FLQP [16]	Beta-casein	102–105
V**LPVP**QKVVP	LPVP	Beta-casein	186–189
FPQYLQ**YPY**Q	YPY [18]	Kappa-casein	79–81
F**LPYPY**YAKPI	LPYPY [18,19]	Kappa-casein	77–81
Y**IPIQY**VLSR	IPIQ [18,19]	Kappa-casein	47–51
Y**IPI**QYVLSR	IPI [16,19,20]	Kappa-casein	47–49
FKN**WV**K	WV [21]	Alpha-lactalbumin	45–46
**WL**PAEYEDGL	WL [22]	Alpha-lactalbumin	123–124
**ILDKVGINY**	ILDKVGINY	Alpha-lactalbumin	114–122
**VGINYWLAHK**	VGINYWLAHK [23]	Alpha-lactalbumin	118–127
**ILDKVGINYWLAHK**	ILDKVGINYWLAHK [23]	Alpha-lactalbumin	114–127
**VAGTWY**SLA	VAGTWY [20]	Beta-lactoglobulin	31–36
TK**IPAVFK**	IPAVFK [20,24]	Beta-lactoglobulin	94–99
**VLVLDTDYK**	VLVLDTDYK [20]	Beta-lactoglobulin	108–116
VL**VLDTDY**K	VLDTDY [24,25]	Beta-lactoglobulin	110–115
LD**IQKVAGTW**	IQKVAGTW [25]	Beta-lactoglobulin	28–35
**IPAVFKIDA**LN	IPAVFKIDA [23]	Beta-lactoglobulin	94–102
TK**IPAVF**K	IPAVF [24]	Beta-lactoglobulin	94–98
TK**IPA**VFK	IPA [6,24]	Beta-lactoglobulin	94–96
QEPVLGP**VR**	VR [16]	Milk Serum albumin	432–433
V**LP**VPQKVVP	LP [16]	Milk Serum albumin	136–137
TK**IP**AVFK	IP [16]	Milk Serum albumin	321–322
F**YP**QLFR	YP [16,18]	Lactotransferrin	185–186
TPDNIDI**WI**GG	WI [22]	Lactotransferrin	144–145

## Data Availability

The original contributions presented in the study are included in the article/Supplementary Material, further inquiries can be directed to the corresponding author.

## Supplementary Materials

The following supporting information can be downloaded at: https://www.mdpi.com/article/10.3390/foods13172721/s1, Table S1: Comparison of that property of synthetic peptides; Table S2: Molecular docking results of ILDKVGINY; Table S3: Molecular docking results of IPDKVGINY; Table S4 Molecular docking results of IIDKVGINY.

## Abbreviations

Dipeptidyl peptidase-IV inhibitor, DPP-IV; CN, Casein; LC-MS/MS, Liquid chromatography-mass spectrometry/mass spectrometry; HPLC, High-performance liquid chromatography; MTT, Methyl thiazolyl tetrazolium; ILDKVGINY, H2N-Ile-Leu-Asp-Lys-Val-Gly-Ile-Asn-Tyr-COOH; IPDKVGINY, H2N-Ile-Pro-Asp-Lys-Val-Gly-Ile-Asn-Tyr-COOH; IIDKVGINY, H2N-Ile-Ile-Asp-Lys-Val-Gly-Ile-Asn-Tyr-COOH; Ile, isoleucine; Pro, proline; Ala, alanine; leu, leucine.
