# Supplementary material for: DPP-IV Inhibitory Peptide against In Vitro Gastrointestinal Digestion Derived from Goat’s Milk Protein and Its Activity Enhancement via Amino Acid Substitution
Source: Foods. 2024 Aug 27;13(17):2721. doi: 10.3390/foods13172721 (PMC11395612; doi:10.3390/foods13172721)
Supplement: Supplementary file 1 [file foods-13-02721-s001.zip › foods-3149336-supplementary.pdf]

Table S1 Comparison of that property of synthetic peptides

Single letter	ILDKVGINY	IPDKVGINY	IIDKVGINY
Multi-letter	H_2N-Ile-Leu-Asp-Lys-Val-Gly-Ile-Asn-Tyr-COOH	H_2N-Ile-Pro-Asp-Lys-Val-Gly-Ile-Asn-Tyr-COOH	H_2N-Ile-Ile-Asp-Lys-Val-Gly-Ile-Asn-Tyr-COOH
Amino acid number	9	9	9
structural formula	$C_{48}H_{79}N_{11}O_{14}$	$C_{47}H_{75}N_{11}O_{14}$	$C_{48}H_{79}N_{11}O_{14}$
Average molecular weight	1034.21	1018.16	1034.21
Accurate molecular weight	1033.58	1017.55	1033.58
Isoelectric point (PI)	9.3	9.3	9.3
Electrostatic charge number at pH=7.0	0.98	0.98	0.98
GRAVY	0.53	-0.07	0.61
Hydrophilic residue ratio	0.09	0.09	0.09
extinction coefficient	1490	1490	1490
Average hydrophilicity	Hydrophobic	Hydrophobic	Hydrophobic

Table S2 Molecular docking results of ILDKVGINY

Binding site	Properties of alkali	Number of keys	bond length
Ser212	hydrogen bond	2	2.72 Å、2.93 Å
Tyr585	hydrogen bond	2	2.42 Å、3.04 Å
Leu214	hydrogen bond	2	2.67 Å、2.85 Å
Trp216	hydrogen bond	2	3.42 Å、3.10 Å
Arg358	hydrogen bond 、Electrostatic force	2、1	2.97 Å、3.18 Å、4.76 Å
Arg356	hydrogen bond	2	3.55 Å、3.35 Å
Ser360	hydrogen bond	1	2.92 Å
Trp305	hydrogen bond	1	3.69 Å、3.37 Å
Thr304	hydrogen bond	1	3.07 Å
Val303	hydrogen bond	1	2.64 Å
Ser209	hydrogen bond	1	2.83 Å
Glu205	hydrogen bond	1	2.64 Å
Pro159	hydrogen bond	1	3.85 Å
Trp154	hydrogen bond	1	4.95 Å
Phe357	hydrogen bond	1	4.57 Å
Ile374	hydrogen bond	1	4.46 Å
Thr156	hydrogen bond	1	3.66 Å

Table S3 Molecular docking results of IPDKVGINY

Binding site	Properties of alkali	Number of keys	bond length
Trp157	hydrogen bond 、Hydrophobic force	1、1	2.76 Å、5.34 Å
Trp154	hydrogen bond 、Hydrophobic force	1、1	3.16 Å、4.63 Å
Trp215	hydrogen bond	2	3.22 Å、3.28 Å
Ser106	hydrogen bond	2	3.01 Å、3.16 Å
Arg61	hydrogen bond 、Hydrophobic force	1、1	3.09 Å、4.86 Å
Thr156	hydrogen bond	2	2.75 Å、2.16 Å
Trp216	hydrogen bond	2	2.50 Å、2.51 Å
Ala210	Hydrophobic force	1	3.85 Å
Pro159	Hydrophobic force	1	4.55 Å
Pro109	Hydrophobic force	1	4.63 Å
Ser212	hydrogen bond	1	3.15 Å
Trp62	hydrogen bond	1	3.15 Å
Ile107	hydrogen bond	1	2.04 Å

Table S4 Molecular docking results of IIDKVGINY

Binding site	Properties of alkali	Number of keys	bond length
Pro159	hydrogen bond 、Hydrophobic force	1、1	3.72 Å、4.05 Å
Arg61	Hydrophobic force	2	3.69 Å、4.40 Å
Pro109	Hydrophobic force	1	3.72 Å
Ser106	hydrogen bond	2	3.10 Å、2.80 Å
Thr156	hydrogen bond	2	2.11 Å、2.92 Å
Ile107	hydrogen bond 、Hydrophobic force	1、1	2.06 Å、4.05 Å
Pro157	Hydrophobic force	1	5.31 Å
Tyr105	Hydrophobic force	1	5.16 Å
Leu69	Hydrophobic force	1	4.87 Å
Ser212	Hydrophobic force	1	1.81 Å
Trp305	Hydrophobic force	1	3.19 Å
His363	Hydrophobic force	1	2.83 Å
Trp216	hydrogen bond	2	2.97 Å、3.75 Å
Glu361	hydrogen bond	2	2.62 Å、3.25 Å
Thr304	hydrogen bond	1	3.11 Å
Trp215	hydrogen bond	1	3.40 Å
Pro218	Hydrophobic force	1	4.43 Å
Ala210	Hydrophobic force	1	5.44 Å
